# A Simple Assay to Screen Antimicrobial Compounds Potentiating the Activity of Current Antibiotics

**DOI:** 10.1155/2013/927323

**Published:** 2013-06-20

**Authors:** Junaid Iqbal, Ruqaiyyah Siddiqui, Shahana Urooj Kazmi, Naveed Ahmed Khan

**Affiliations:** ^1^Department of Biological and Biomedical Sciences, Aga Khan University, Stadium Road, Karachi 74800, Pakistan; ^2^Department of Microbiology, University of Karachi, Karachi 75270, Pakistan

## Abstract

Antibiotic resistance continues to pose a significant problem in the management of bacterial infections, despite advances in antimicrobial chemotherapy and supportive care. Here, we suggest a simple, inexpensive, and easy-to-perform assay to screen antimicrobial compounds from natural products or synthetic chemical libraries for their potential to work in tandem with the available antibiotics against multiple drug-resistant bacteria. The aqueous extract of *Juglans regia* tree bark was tested against representative multiple drug-resistant bacteria in the aforementioned assay to determine whether it potentiates the activity of selected antibiotics. The aqueous extract of *J. regia* bark was added to Mueller-Hinton agar, followed by a lawn of multiple drug-resistant bacteria, *Salmonella typhi* or enteropathogenic *E. coli*. Next, filter paper discs impregnated with different classes of antibiotics were placed on the agar surface. Bacteria incubated with extract or antibiotics alone were used as controls. The results showed a significant increase (>30%) in the zone of inhibition around the aztreonam, cefuroxime, and ampicillin discs compared with bacteria incubated with the antibiotics/extract alone. In conclusion, our assay is able to detect either synergistic or additive action of *J. regia* extract against multiple drug-resistant bacteria when tested with a range of antibiotics.

## 1. Introduction

Bacterial infections result in 17 million deaths worldwide annually, mostly in children and the elderly [1]. The morbidity and mortality associated with bacterial infections have remained significant, despite advances in antimicrobial chemotherapy. The situation has worsened with bacterial resistance increasing to available antibiotics. From the 1930s to 1960s, twenty classes of antibiotics were marketed but in the last four decades only three new classes of antibiotics have been introduced [1–5]. Today, multiple drug-resistant bacteria such as methicillin-resistant *Staphylococcus aureus* (MRSA), vancomycin-resistant enterococci (VRE), *Acinetobacter baumannii*, *Klebsiella pneumonia*, *Escherichia coli*, and *Pseudomonas aeruginosa* pose a serious threat to human and animal health. There is also the emergence of bacterial pathogens with intrinsic resistance to antibiotics making the available antibiotics obsolete [2]. A feasible approach to counter such a crisis is to revive the use of available antibiotics against multiple drug-resistant bacteria by improving their efficacy. Here we suggest a simple, inexpensive and easy assay to perform screening of antimicrobial compounds from natural products [1] such as *Juglans regia* or synthetic chemical libraries for their potential to work in tandem with the available antibiotics against multiple drug-resistant bacteria. The basis of this assay is that the extract may enhance the killing effect of synthetic antibiotics by overcoming the resistant phenotype. 

## 2. Materials and Methods

### 2.1. Bacterial Isolates

Two clinical isolates of *Salmonella typhi *and enteropathogenic *E. coli* were used in this study. *E. coli *isolate was derived from diarrheal stool sample and that of *Salmonella typhi* was obtained from the blood cultures of a typhoid patient. The clinical samples were plated on blood agar plates. All the bacterial isolates were characterized by colony morphology and biochemical testing and their identity confirmed by using API-20E system (bioMérieux). Both isolates were preserved in glycerol stocks. For both bacteria, antibiotic evaluation test was performed on Mueller-Hinton agar using Kirby-Bauer method as per Clinical Laboratory Standards Institute [6, 7]. *Salmonella typhi *was considered multiple drug resistant, if it exhibited growth on antibiotic disks containing ampicillin 10 *μ*g (breakpoint inhibition zone for ampicillin for *S. typhi* was considered 14 mm), cefuroxime 30 *μ*g (breakpoint inhibition zone for cefuroxime for *S. typhi* was considered 18 mm), and aztreonam 10 *μ*g (breakpoint inhibition zone was considered 24 mm) as per EUCAST guidelines. Similarly, *E. coli *was considered multiple drug resistant, if it exhibited growth on antibiotic disks containing ampicillin 10 *μ*g (breakpoint inhibition zone for ampicillin for *E. coli* was considered 14 mm), cefixime 5 *μ*g (breakpoint inhibition zone was considered 17 mm), and aztreonam 10 *μ*g (breakpoint inhibition zone was considered 24 mm). 

### 2.2. *Juglans regia* Extract

The aqueous extract of *J. regia *(commonly known as Persian walnut or English walnut tree) bark was prepared by taking 10 gram bark and mixing it in 100 mL water overnight. Next day, the suspension was incubated in boiling water bath for two minutes. Subsequently, the mixture was cooled, centrifuged at 2500 ×g, and the supernatant was filtered through 0.2 *μ*m filter. The filtrate was evaporated using a refrigerated CentriVap Concentrator (Labconco Corp.) evaporator until dry and stored at 4°C until further use. 

### 2.3. Simple Plating Assay

The proposed assay utilizes the modified version of the antibiotic disc diffusion assay (“Kirby-Bauer” assay) [6, 7]. The Mueller-Hinton agar plates were prepared containing aqueous extract of *J. regia* (final concentration of extract was 0.625 mg per mL of agar). Next, 20 mL of the overnight grown culture of multiple drug-resistant bacteria, *S. typhi* and *E. coli,* was poured on Mueller-Hinton agar containing *J. regia* extract plates and left for 5 min. Following this incubation, the excess bacterial cultures were aspirated and plates were left in the biosafety hood to dry. Next, filter paper discs impregnated with different antibiotics (aztreonam, cefuroxime, ampicillin, and cefixime) were placed on the agar surface and plates were incubated at 37°C overnight. Any change in the diameter of the inhibition zone was measured to determine bacterial sensitivity as per Clinical Laboratory Standards Institute [7]. Any increase in the diameter of the inhibition zone was investigated to determine whether a specific antibiotic plus *J. regia* extract has the potential to target the multiple drug-resistant bacteria. For controls, *S. typhi* and *E. coli* were plated on Mueller-Hinton agar plates containing extract but without antibiotic discs. In some experiments, *S. typhi* and *E. coli* were plated on Mueller-Hinton agar plates without extract but with antibiotic discs. 

## 3. Results 

### 3.1. Aqueous Extract of *J. regia* Has Either a Synergistic Or Additive Action Against Multidrug-Resistant (MDR) Bacteria When Tested with a Range of Antibiotics

When plated on Mueller-Hinton agar plates containing *J. regia* extract but without antibiotic discs, the growth of neither *S. typhi* nor *E. coli* was affected. However, in the presence of antibiotics, the results showed that the zone of inhibition for *S. typhi *around the aztreonam antibiotic disc increased from 20 mm in Mueller-Hinton agar plates to 29 mm in Mueller-Hinton agar containing *J. regia* extract plates. Thus, the addition of *J. regia* extract apparently renders once-resistant *S. typhi* strain sensitive to aztreonam again (breakpoint inhibition zone diameter of ≤24 mm was considered resistant) (Table 1). Similarly, the zone of inhibition around the ampicillin antibiotic disc increased from 13 mm in Mueller-Hinton agar plates to 19 mm in Mueller-Hinton agar containing *J. regia* extract plates (Table 1). Thus, the addition of *J. regia* extract apparently renders once-resistant *S. typhi* strain sensitive to ampicillin again (breakpoint inhibition zone diameter of ≤14 mm was considered resistant) (Table 1). However, *J. regia* had limited effect on the resistance of *S. typhi* strain to cefuroxime. The zone of inhibition around the cefuroxime antibiotic disc increased from 12.5 mm in Mueller-Hinton agar plates to 18 mm in Mueller-Hinton agar containing *J. regia* extract plates (breakpoint inhibition zone diameter of ≤18 mm was considered resistant) (Table 1). 

The zone of inhibition for enteropathogenic *E. coli* around the ampicillin antibiotic disc increased from 13 mm in Mueller-Hinton agar plates to 18 mm containing *J. regia* extract plates. Thus, the addition of *J. regia* extract renders once-resistant *E. coli *strain sensitive to ampicillin again (breakpoint inhibition zone diameter of ≤14 mm was considered resistant) (Table 1). Similarly, the zone of inhibition around the cefixime antibiotic disc increased from 16 mm in Mueller-Hinton agar plates to 22 mm in Mueller-Hinton agar containing *J. regia* extract plates (Table 1). Thus, the addition of *J. regia* extract renders once-resistant *E. coli* strain sensitive to cefixime again (breakpoint inhibition zone diameter of ≤17 mm was considered resistant) (Table 1). However, *J. regia* extract had limited effect on the resistance of *E. coli *strain to aztreonam. The zone of inhibition around the aztreonam antibiotic disc increased from 14.5 mm in Mueller-Hinton agar plates to 21 mm in Mueller-Hinton agar containing *J. regia* extract plates (breakpoint inhibition zone diameter of ≤24 mm was considered resistant) (Table 1). Both *E. coli* and *S. typhi* lawns grew normally on plates added with *J. regia's* extract without antibiotics discs (data not shown).

## 4. Discussion

The basis of the proposed assay is that natural products with potential antibacterial activity overcome the resistance phenotype of multiple drug-resistant (MDR) bacteria and render them susceptible to the available antibiotics. The proposed assay has the potential to test the efficacy of various antibiotics on a single plate in an easy-to-use manner. In addition, the proposed assay is cost-effective and can be performed in a basic microbiology laboratory. Bacteria employ a variety of strategies to develop resistance against a single or group of antibiotics. These include drug-degrading and -modifying enzymes, reduced membrane permeability for drugs, expression of drug efflux pumps, overproduction and alteration of drug targets, and bypassing the inhibitory pathway [2]. Thus, strategies (or use of natural products) that can block the aforementioned drug resistance mechanisms will allow the clinical use of currently available antibiotics. This is not a novel concept and several studies have reported the use of multiple compounds to produce synergistic or additive effects against microbial pathogens [3, 6, 8, 9]. One such example is coamoxiclav, a combination of amoxicillin and clavulanic acid. This combination is also effective against amoxicillin-resistant bacteria because clavulanic acid is an inhibitor of β-lactamase, an enzyme which degrades β-lactam drugs including amoxicillin. Other similar combinations which are in clinical use include ampicillin/sulbactam and piperacillin/tazobactam [10]. The findings reported here suggest that *J. regia* extract can extend the clinical use of available antibiotics. This is consistent with previous findings which suggest that *J. regia* bark extract has either synergistic or additive action against Gram-negative bacteria [11]. However, the mechanisms of action remain unknown, and future studies are needed to identify the substance(s) present in the *J. regia* extract responsible for the synergy with β-lactams. Notably, *J. regia* is commonly used in some countries as a toothbrush, and it has been suggested that brushing teeth with this bark may improve oral hygiene, prevent plaque and caries formation, and reduce the incidence of gingival and periodontal infections [12]. Although there is no toxicity data available for *J. regia*, recent findings suggest that *J. regia* extract treatment (150 mg/kg) resulted in protective restoration of decreased antioxidants in a murine urotoxicity model [13], albeit at considerable lower concentrations compared with the present study. 

It is instructive to point out that many molecular biosystems and biomedical systems belong to the multilabel systems in which each of the constituent molecules possesses one or more than one function or feature and hence needs one or more than one label to indicate its attribute(s), as observed in recent antimicrobial peptides research [14]. Antimicrobial peptides (AMPs) belong to the class of well-studied multifunctional molecules. They are part of innate immune mechanisms, found in almost all cellular organisms. Many AMPs have been shown to possess a broad spectrum of activities against viruses, bacteria, fungi, protists, parasites, and surprisingly, even against tumor cells [15]. Mostly, AMPs are cationic peptides and work through interfering with cell membrane integrity or enter into the target cell and inhibit vital cellular processes like DNA replication, transcription, translation, and metabolic pathways [15]. Other important features of AMPs include their low toxicity to mammalian cells and slow rate of emergence of bacterial resistance against them in comparison with the available antibiotics [16]. Recently, a number of powerful computational tools have been developed to predict or design AMPs bearing one or more activities [17, 18]. All of the above features have made AMPs a promising candidate for future therapeutics [15]. However, as a first step, here we have tested the single-label system. Many studies have indicated that cheminformatics [19], mutagenesis [20], molecular docking [21, 22], predicting drug-target interaction [23], and bioinformatics [24, 25] can timely provide very useful information and insights for drug development and hence are widely welcomed by the scientific community. The present study attempted to develop a novel method to screen potential antimicrobials using plate assay, hoping that the new method can become a useful tool for drug development.

Overall, our findings provide support that *J. regia* extract enhances the efficacy of tested antibiotics against multiple drug-resistant bacteria. The proposed assay can easily be used to screen a wide range of natural products and compound libraries. Our assay has an advantage of screening a pure compound or natural product against five to eight different antibiotics on the same plate. Because of its cost effectiveness and simplicity, this assay could be used by labs which have just basic microbiology setup and limited funding and allow them to contribute in the discovery of potential antimicrobials that target drug-resistant pathways in multiple drug-resistant bacteria, the mechanisms of which will be explored in further studies. 

## Figures and Tables

**Table 1:** *Juglans regia* extract helped enhance the efficacy of synthetic antibiotics and overcame the resistant phenotype. Data are presented as the mean ± standard error of three independent experiments.

Antibiotic	Zone of inhibition, mm (antibiotic alone)	Zone of inhibition, mm (antibiotic + *Juglans regia* extract)	*P* value*
Aztreonam (MDR *Salmonella typhi*)	20 ± 3.4	29 ± 6.2	<0.01
Cefuroxime (MDR *Salmonella typhi*)	12.5 ± 2.2	18 ± 2.5	<0.01
Ampicillin (MDR *Salmonella typhi*)	13 ± 2.5	19 ± 1.8	<0.001
Aztreonam (MDR enteropathogenic *E. coli*)	14.5 ± 3.5	21 ± 5.3	<0.01
Cefixime (MDR enteropathogenic *E. coli*)	16 ± 5.1	22 ± 4.7	>0.05
Ampicillin (MDR enteropathogenic *E. coli*)	13 ± 0.9	18 ± 3.1	>0.01

*P values were calculated using one-tailed paired *t*-test.
